# Delta-5^®^ oil, containing the anti-inflammatory fatty acid sciadonic acid, improves skin barrier function in a skin irritation model in healthy female subjects

**DOI:** 10.1186/s12944-022-01643-9

**Published:** 2022-04-20

**Authors:** Alvin Berger

**Affiliations:** SciaEssentials, LLC and Sciadonics, Inc, 1161 Wayzata Blvd E Unit 30, MN 55391 Wayzata, United States

**Keywords:** Barrier function, Epidermis, Irritant, Sciadonic acid, Skin, SLS, Sodium lauryl sulfate, Stratum corneum, TEWL, Transepidermal water loss

## Abstract

**Background:**

Sciadonic acid (SA) is an anti-inflammatory fatty acid displacing arachidonic acid (ARA) from specific phospholipid pools, thus modulating downstream pro-inflammatory lipid mediators. Its novel anti-inflammatory actions have been studied in vitro, in pre-clinical models, and stemming from testimonials, after topical- and oral application. It has not been tested in a formal clinical study for topical benefits previously. Skin barrier layer was our focus as it has a critically important role in maintaining skin moisture balance.

**Methods:**

Herein, forearm skin was left undamaged; or barrier layer was chemically-damaged with 2% sodium lauryl sulfate (SLS) for 24 h. SLS-damaged skin was left untreated or treated with Delta-5® oil containing 24% SA twice daily for 27 days. Barrier function was assessed by open chamber transepidermal water loss (TEWL) and skin surface impedance on days 0 (clear skin), -1 (1-day post-SLS), -2 (2-days post-SLS, 1-day post-Delta-5), -3, -7, and − 28.

**Results:**

Relative to day 1, Delta-5 oil statistically significantly decreased TEWL vs. untreated damaged sites, on days 3 (125% more reduced), -7 (74% more reduced), and − 28 (69% more reduced). Decreases in TEWL following chemical damage indicates improved skin barrier repair and healing. Similar patterns were quantified for skin impedance. There was also reduced redness observed on days 3 and − 7 with Delta-5 oil vs. untreated SLS-damaged skin.

**Conclusions:**

Delta-5 oil thus has anti-inflammatory potential in human skin, under controlled clinical conditions, to accelerate irritant-induced healing, and improve skin barrier function. Improvement in barrier function would benefit dermatitis, acne, eczema, and skin scarring. In normal skin, Delta-5 oil has potential to promote healthy, moisturized skin; and improve skin structure, elasticity, and firmness.

## Background

### Sciadonic acid (SA)

**Fig. 1:**
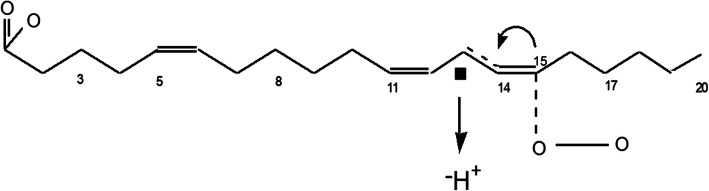
Structure of sciadonic acid (SA). SA is a non-methylene interrupted fatty acid present in Delta-5 oil as a triacylglycerol, representing 20–25% of total fatty acids in the oil. It is a 20-carbon fatty acid, with 3 double bonds present in Δ-5, 11, and 14 positions in cis-configuration. As a result of having only 2 methylene-interrupted fatty acids, it has good oxidative stability like a dienoic fatty acid. In skin, sciadonic acid exerts anti-inflammatory properties by displacing arachidonic acid from lipid pools; but may also potentially form bioactive, aborted cyclooxygenase and lipoxygenase and other eicosanoid products (a very poorly studied area). In our example herein, the C13-hydrogen at the center of the 1,4 cis, cis pentadiene system is extracted, following oxygen attack at C-15, forming 15-hydroxy-5,11,13-eicosatrienoic acid by the cyclooxygenase system. Another cyclooxygenase product (not shown) is 11-hydroxy-5,12,14-eiosatrienoic acid [64]. If SA were acted on by a skin 12-lipoxygenase, then 12-hydroxy-5,10,14-eicosatrienoic acid would be theoretically formed (not shown). See the "Introduction" section for further discussion and references

SA (all cis Δ5,11,14 eicosatrienoate) is principally found in various gymnosperm plant species. Its structure and example of an eicosanoid metabolite are shown in Fig. 1. SA is an anti-inflammatory fatty acid analog of arachidonic acid (5,8,11,14 eicosatetraenoate; ARA) lacking the Δ-8 double bond necessary for formation of typical pro-inflammatory, fully-formed, eicosanoid mediators. Our group and others have shown SA to exert anti-inflammatory actions by reducing levels of ARA and downstream mediators in *in vitro* and animal models [1–8]. Topical anti-inflammatory properties using lipidic seed extracts rich in SA have also been demonstrated [1, 6, 9–11]. Delta-5® oil (Delta-5) refers to the oil sold by SciaEssentials®, LLC with 20–25% SA. Delta-5 was used herein; and Delta-5 or a similar oil was used in some of our previous experiments as described below.

In addition to the anti-inflammatory benefits and other physiological benefits of SA (see references in previous paragraph), SA is relatively oxidatively stable. When there are two or more double bonds present in a methylene interrupted arrangement in a fatty acid molecule, in response to oxidative stress, the fatty acyl molecules are subject to hydrogen abstraction on the carbon flanked by two double bonds, and subsequent peroxidation (autoxidation). In the case of SA, only two double bonds (Δ11–12 and Δ14–15) are methylene-interrupted and subject to peroxidation. This could contribute to greater stability of SA relative to a typical triene, in which all three double bonds are methylene-interrupted (e.g., alpha linolenic acid present in linseed oil). SA can thus be added to skin formulations and food products (if consumed orally) in commercial products requiring longer shelf-lives; and with less complicated and less expensive antioxidant systems mandated.

We previously demonstrated that topically-applied methyl esters from an oil similar to Delta-5 led to SA incorporation into mouse ear phospholipids, including the important signaling phosphatidylinositol pool; and reduced phospholipid levels of ARA; and -ARA- and TPA (12-*O*-tetradecanoylphorbol-13-acetate) -induced mouse ear edema [1]. Purified SA taken up by cultured human skin keratinocytes reduced ARA, and -pro-inflammatory prostaglandin E_2_ (PGE_2_) levels dramatically. SA could thus be an important topical anti-inflammatory therapeutic ingredient or pharmaceutical agent.

SA could also de-age skin and improve skin structural integrity. In human tissue fragments derived from elective blepharoplasty surgery from healthy volunteers, there was a 28% increase in Collagen Type 1 synthesis vs. active placebo (mean square pixels increased from 14,462- to 17,846 units; *p* < 0.0001); and a larger 49% increase relative to control/basal condition (from 12,000- to 17,846 units; *p* < 0.05), with 3% Delta-5 after 72 h incubation (unpublished results).

Delta-5 also has topical photoprotective properties. In human dermal fibroblasts, Delta-5 enhanced cell viability vs. vehicle control following ultraviolet UVA radiation (viability increased 3.8-fold from 13- to 49%; *p* < 0.05, *n* = 3, 2-tailed *t*-test) [12]. There were no effects on UVB-induced cell damage. Delta-5 (alone or in synergy with other approved ingredients) could be used after sunburns to promote accelerated healing of skin, consistent with client testimonials.

In addition to experimental results, testimonials from customers, dermatologists, medical doctors, and aestheticians using Delta-5 have observed rapid benefits to their skin including reductions in redness and wrinkling, and collagen boosting. Professional beauty channel customers have observed more rapid- and less painful healing following aesthetic treatments with lasers, fillers, peels, and masks.

### SLS irritant-induced skin barrier injury model

The skin barrier layer has the most important role in maintaining skin moisture balance. The barrier layer/extracellular domain of the stratum corneum is composed of lipids, predominately ceramides, free fatty acids, and cholesterol, in a precise lipid lamellar organization [13, 14]. We envisioned SA could benefit barrier function by decreasing skin inflammatory tone; possibly other mechanisms could be involved. Sodium lauryl sulfate (SLS; also known as sodium dodecyl sulfate) is a type of concentrated soap/surfactant/detergent with an anionic hydrophilic polar group [15]. SLS is used in acute- and cumulative irritation skin barrier injury models; and in acute irritant contact dermatitis- and inflammation models [16]. SLS was chosen as an irritant because of its ability to penetrate and impair the skin barrier [17]. SLS is typically applied once at 1–2% for 24 h (2% for 24 h was used herein); or repeatedly at lower concentrations (e.g., 0.1%) over 3 weeks [18]. At 2%, SLS can induce localized hyperalgesia and inflammation, and release pro-inflammatory pain mediators [19]; inflammation can last up to 6 days during the irritation period.

### TEWL, skin impedance and redness

Transepidermal water loss (TEWL) is the most important objective measurement for assessing barrier function and skin integrity in healthy individuals, and those with skin diseases associated with skin barrier dysfunction. TEWL is measured noninvasively with an evaporimeter probe to detect the quantity of low-level condensed water diffusing/transpiring across a fixed area of stratum corneum skin surface per unit time [20, 21]. Higher TEWL is associated with skin barrier impairments and damaged and irritated stratum corneum and epidermis, as seen in unhealed wounds, in skin scarring, in response to food allergens, and in psoriasis, eczema, and particularly atopic dermatitis [22]. Lower TEWL is associated with healthy skin, and improved skin barrier- function and structure. A reduction in TEWL generally indicates proportionately higher film (barrier) integrity and/or wound closure in wound healing studies. We assessed SLS-induced changes to skin by measuring TEWL.

Damage to the stratum corneum outer skin layer can lead to increased surface water. This was measured noninvasively by surface impedance as electroconductivity, an indirect measure of the retained water content of the skin as a function of the skin’s dielectric value. Skin impedance can be measured alongside TEWL to compare the two techniques and gain additional confidence in results [17, 23–25].

Visual assessment of skin reactions and color has long been used to evaluate the safety of chemicals and preparations that contact skin [26]. Skin redness was evaluated by Visual Expert Grading, on a scoring scale of 1–10, following high resolution photography of test sites.

This study represents the first time sciadonic acid (here, as a 24% fatty acid component of Delta-5) was formally tested in any controlled clinical setting, for topical or oral use. The primary goal of this first pilot study was to determine if Delta-5 treatment could improve irritant-induced barrier damage relative to damaged skin without any other treatment. Specifically, our pilot study with 7 subjects was conducted to test if Delta-5 could reduce TEWL, impedance, and redness in the well-established SLS irritation injury model. We also report results on stability and repeat insult patch test safety studies, which were clinical prerequisites for conducting the SLS study.

## Methods

### Test materials all studies

The starting plant seed source was from *Nageia nagi* seeds (China), used for preparation of our commercial Delta-5® oil currently sold by SciaEssentials® (INCI name: Nageia Nagi Seed Oil). Seeds were sun-dried, tempered, and cracked to partially remove the hulls and skins surrounding the nut “meat”. This material was pressed, filtered, then refined, bleached, deodorized, and winterized (RBDW) to obtain the final cosmetic-grade oil. The oil was supplemented with mixed natural tocopherols (Dadex GT-2 NGM – Food, Mississauga, ON, Canada) to a final concentration of 1500 mg Dadex GT-2/kg oil, then stored under nitrogen at -20 °C in 10 mL brown dropper bottles until use. Main fatty acids present (as % total fatty acid methyl ester peaks identified by gas chromatography) were 15.8% oleic acid (18:1n-9), 42.3% linoleic acid (18:2n6), 11.4% eicosadienoic acid (dihommolinoleic acid; 20:2n-6); and 24.4% SA. The fatty acids are present almost exclusively bound to glycerol as triacylglycerols based on our analyses and published literature. Oil was of high quality for skin applications based on color lack of odor, and low viscosity, with 0.11% free fatty acids and peroxide value of 0.66 mEq/kg. Levels of microbes (Salmonella, *E. Coli*, and yeast and mold) and heavy metals (arsenic, cadmium, mercury, lead) were negative or within acceptable limits. Oil extractions and refinement, and analytical testing were performed by our commercial partners using accredited laboratories and procedures. Prior to experimentation, oils were thawed at room temperature.

### Stability testing study methods and results

Prior to safety and clinical testing, stability testing was performed. Open vessels containing three different lots of Delta-5 oil were placed in a controlled temperature chamber at 25- and 40 °C for 3 months, with assessments for changes in color, odor (due to fatty acid oxidation), and homogeneity of appearance (due to precipitation). Oils were evaluated initially, and at weeks 1, 2, 3, 4, 8, and 12. At both temperatures, and at all time points, there were no changes observed in color and odor; and the oils remained homogeneous (pictures not shown). For personal care testing, 3 months stability at 40 °C is generally equated with 1 year stability at 25 °C. For an oil in a neat formulation with only exogenous tocopherols added as an additional antioxidant, and up to 78% polyenes (dienes and trienes), this represents very good stability in an open system, not nitrogen gas-blanketed to prevent lipid oxidation. In our SA-rich oils kept at -20 °C under nitrogen, we have not observed changes in fatty acid content nor peroxide value after 3 years storage. As described in the Introduction section, although SA is a triene, its oxidative stability is more like that of a more stable diene, due to the non-methylene interrupted arrangement of the three double bonds. This factor is likely responsible for the prolonged stability observed in this preliminary stability investigation.

### Repeat insult patch test study methods and results

A standard Repeat Insult Patch Test (RIPT) was conducted to verify Delta-5 oil was not a contact sensitizer nor skin irritant [27]. The study was conducted from 4/5–5/12/21 at Advanced Science Laboratories, Inc. (New City, NY) following Standard Operating Procedures with modifications for our non-viscous oil, in compliance with Institutional Review Board guidelines (CFR Title 21 Part 56, Subparts A, B, C, D). Evaluators were required to pass a visual discrimination examination overseen by a Board-Certified Ophthalmologist using the Farnsworth-Munsell 100 Hue Test, which determines a person’s ability to discern color against a black background. This test was additionally modified to include a flesh tone background approaching actual use conditions; erythematous skin was graded according to intensity.

### Inclusion and exclusion criteria for RIPT

Inclusion criteria consisted of being free of any dermatological or systemic disorder; free of any acute or chronic disease; in general good health; willing to complete a preliminary medical history; willing to sign an informed consent; willing to cooperate with the research team; willing to have test materials applied; and willing to complete the full course of the study. Exclusion criteria consisted of being under 18 years of age; currently taking topical or systemic medication that could mask or interfere with test results; having a history of acute or chronic disease that might interfere with or increase risk associated with study participation; having chronic skin allergies; and being pregnant or lactating.

### Recruitment and subjects for RIPT

Participants were recruited by advertisements in local periodicals, community bulletin boards, phone solicitation, electronic media, or combinations thereof. There were 63 subjects enrolled, and 53 completers. This represents a typical percentage (84%) of completers for RIPT testing, which involves multiple patch applications and site visits for scoring. Dropouts resulted from subjects being unreliable, developing COVID-19, and for other reasons, unrelated to Delta-5 per se. The age range was 19–74, with 15 males, and 39 females. There were 32 Caucasians, 15 Hispanics, and 7 Blacks.

### Testing equipment for RIPT

Equipment consisted of 2 cm X 2 cm gauze patches, covered with occlusive tape (the assemblage was custom made by Strukmyer, LLC, Mesquite, TX); and 1 mL volumetric syringes without needles to dispense Delta-5. Subjects were requested to bathe or wash as usual before arrival at the facility. Delta-5 was refrigerated, then prior to use, equilibrated at room temperature. Delta-5 (0.2 mL) was dispensed onto patches, and patches applied directly to infrascapular regions of the back, to right or left of midline. Subjects were instructed to not wet or expose test areas to direct sunlight. After 24 h, patches were removed by panelists at home, and fresh patches with Delta-5 re-applied at home. This procedure was repeated for at least nine 24-h induction phase exposures.

### Scoring and results for RIPT

Reactions were scored just before applications 2–9; and at the re-test date following application 9 at 24- and 48 h after patch removal. Comparisons were made between the 9 inductive responses and the retest doses. At the study conclusion, a consulting Dermatologist confirmed the Study Director’s conclusions. The scoring scale ranged from 0.0 (no evidence of any effect) to 4.0 (severe deep red erythema with vesiculation or weeping), with intermediary scores with defined characteristics of 0.5, 1.0, 2.0, and 3.0. Amongst the 63 subjects initially enrolled and the 53 completers, evaluators scored all sites as 0.0, indicating Delta-5 was not a contact irritant nor skin sensitizer. There were also no observed adverse reactions demonstrating erythema and/or edema. Having demonstrated good stability, and lack of irritancy and skin sensitization, we proceeded to our skin irritation barrier function testing, described below.

### Transepidermal water loss and impedance study methods and skin redness methods

 The study was conducted from 6/14 − 7/21/21 at Advanced Science Laboratories (New City, NY) following Standard Operating Procedures with modifications for our non-viscous Delta-5 oil, in compliance with Institutional Review Board guidelines (CFR Title 21 Part 56, Subparts A, B, C, D).

### Inclusion and exclusion criteria; recruitment and subjects

Inclusion and exclusion criteria, informed consent, and recruitment were as described for the Repeat Insult Patch Test. For TEWL testing, an additional inclusion criterion was that subjects abstain from using moisturizing products on the volar forearm, for at least two days prior to study commencement. There were 7 subjects enrolled, and 7 completers, monitored for 28 d, across three sites on the skin. The 7 subjects were coded, 56 0949, 48 9460, 39 1287, 68 2278, 70 9478, 56 3379, and 58 9750. The age range was 45–58; and all were female and Caucasian.

### Testing equipment

Bioengineering parameters were assessed by TEWL and impedance. TEWL was measured with an open-chamber DermaLab System computerized Evaporimeter (cyberDERM, Inc., Cortex Technology, PA), equipped with a built-in analog-to-digital (A/D) converter for sending data streams to the host computer via a serial interface. TEWL readings were conducted in a quiet area, apart from general traffic and test center activity. Readings were obtained by placing the probe lightly in contact with the skin surface. Measurements of cutaneous evaporation rate are expressed as g/m^2^h. TEWL measurements require about 1 min to allow for equilibrium in the chamber.

Skin surface water was measured by surface impedance as electroconductivity with a Novameter (Nova Dermal Phase Meter, Model DPM 9003, Nova, Technology Corp., Gloucester, MA). The meter provides a relatively, indirect measure of skin retained water content as a function of the skin’s dielectric value (see novatechcorp.com/dpm.html for a more technical explanation). Skin impedance was recorded automatically when equilibrium was achieved, which is typically after 5 measurements in the same spot. Units are in arbitrary Dermal Phase Meter impedance units (DPMIU).

Skin redness was evaluated by colorimetric Visual Expert Grading (VEG) measurement from high resolution photography (photographs not shown), on a scoring scale of 0–10 (VEG Score; VEGS), utilizing equipment and techniques employed at Advanced Science Laboratories. The camera does not touch the skin. A score of 0 represents no redness and clear skin; a score of 10 represents maximal redness possible. Redness scores were evaluated based on values at each time point, with- and without subtracting D0 and D1 values. Redness was also assessed by summing the counts across categorical scores 0, > 0, 0–1, 2–4, and 5–7 (7-the highest score observed).

### Experimental design

**Fig. 2:**
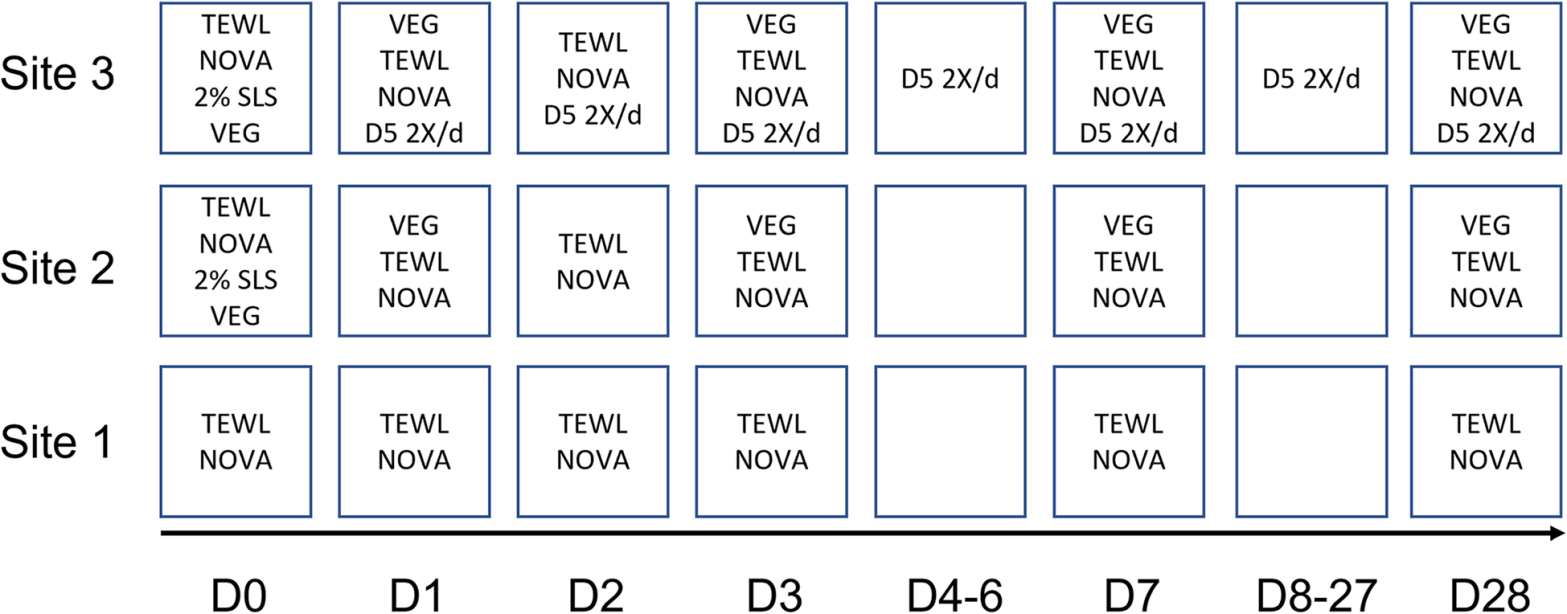
Simplified Experimental Design. Shown are measurements and treatments applied to each subject’s forearm on days 0–28, in the order conducted (top of each box, down). Test site 1, Untreated control; Site 2, Untreated control, chemically damaged with 2% SLS; and Test Site 3, Delta-5 oil applied to SLS-damaged skin. TEWL (g/m^2^h) and NOVA Impedance (units are in arbitrary Dermal Phase Meter impedance units; DPMIU) were measured just before SLS was applied to the skin on Day 0 (baseline); and redness (measured by Visual Expert Grading Scores; VEGS) was measured just after SLS was applied to the skin on Day 0*. On Day 1, redness, TEWL and impedance values are measured 1 day after SLS injury, then Delta-5 oil is applied to the Delta-5 oil group, so that on Day 2, Delta-5 oil has been on the skin for 24 h. Redness was not measured on Day 2. D5 2X/d, Delta-5 oil applied twice per day (see text for further details)

Panelists first reported to the testing facility with forearms devoid of topical treatments. Subjects were acclimated to ambient environment 15 min minimum prior to biophysical measurements. The acclimation procedure was repeated for each subsequent evaluation time point. All subjects received verbal instructions regarding product use and study restrictions. Subjects were required to apply 3 drops (about 27 mg/drop) of Delta-5 twice daily (162 mg total in the 6 drops/day). Each 3-drop application spread into a circle with diameter of 15–20 mm. Designated test site areas were midway between wrist and elbow; and left or right inner volar forearm regions. Assignment of Delta-5 to left or right volar forearm was randomized. Test Site 1 served as untreated, undamaged control; Test Site 2 served as Untreated, SLS-damaged skin; Test Site 3 served as Delta-5 treatment applied to SLS-damaged skin. Delta-5 oil was received refrigerated, then prior to first use, equilibrated at room temperature, and kept at room temperature for the remainder of the experiment. The timing for measurements of TEWL, impedance and redness are explained below and in Fig. 2.

Day 0 (baseline): Baseline TEWL and impedance measurements were obtained from test sites 1–3 just before SLS-damage and initial Delta-5 application. The order of measurements was TEWL, impedance, then redness. Redness was measured on D0, but just after SLS was applied to Sites 2 and 3; and is designated D0*. SLS (0.2 g) was diluted to a 2% final concentration in distilled water, dispensed onto an occlusive, hypoallergenic patch and applied one time only directly to skin (Sites 2 and 3).

Day 1 (1 d after SLS): Patches were removed about 24 h post-application of 2% SLS and discarded. The order of measurement was redness (Sites 2–3), TEWL (Sites 1–3) and then impedance (Sites 1–3). After TEWL measurement, Delta-5 was applied to Site 3 twice daily each morning and before bedtime, from Day 1-Day 28.

Days 2 (i.e., 2 d after SLS, 1 d after Delta-5 oil): TEWL and impedance measured at Sites 1–3. Redness was not assessed. Delta-5 oil was applied to Site 3 twice daily on Days 1–28.

Days 3, 7, and 28: Redness (Sites 2–3), TEWL (Sites 1–3) and then impedance (Sites 1–3) were measured. Delta-5 oil was applied to Site 3 twice daily on Days 1–28.

### Statistics

Data were first evaluated for normal distributions and equal variances. *p*-Values generated from Shapiro-Wilk Test on TEWL and impedance differences between days (D2-D1, D3-D1, D7-D1, and D28-D1) were > 0.01 indicating normal distribution, so data were evaluated with parametric testing with no data rank transformation. Effects of SLS alone (prior to application of Delta-5) were evaluated by comparing D1-D0 with 1-tailed paired *t* tests (1-tailed since SLS is known a priori to increase TEWL and impedance). Technicians consistently applied Delta-5 to the more SLS-damaged site (Site 3); this was verified by evaluating D1-D0 TEWL values for sites to be treated with Delta-5 and untreated sites. Two-tailed *t* tests were used to test for temporal changes in the control site (no SLS). Following SLS damage, to determine if Delta-5 improved healing over the non-treated SLS sites, we evaluated differences between treated and untreated sites by evaluating D2-D1, D3-D1, D7-D1, and D28-D1, using repeated measures analysis of variance (ANOVA), with 1-tailed, paired testing of *p*-values (one tailed because Delta-5 oil is known to expedite healing based on a priori data). As there were changes in TEWL in the control group for D1-D0, a similar repeated measures ANOVA statistic was generated after adjustment for control values, by dividing values collected at each day by the corresponding control value. This correction resulted in a lower *p*-value for D3-D1, a time point at which there was more variability; variability was also reduced for other time points. Our main focus was thus on the control-corrected values for TEWL. Impedance values were evaluated as differences relative to D1, with and without correction for control values; results are shown without control-correction due to high variability in the control. For skin redness, similar statistical conclusions were reached with parametric *t *testing and with Wilcoxon signed rank testing; so only parametric statistics are described for simplicity and consistency with the statistical approaches used for the TEWL and impedance evaluations. Redness was evaluated at each time point; and the data are also described as categorical variables. Statistics were mainly generated using SAS version 9.4 (SAS Institute, Inc., Cary, NC). Where appropriate, if not indicated otherwise, results in the text represent means ± 1 standard deviation (SD).

## Results SLS study

### TEWL-control skin and SLS treatment alone

TEWL increased in control skin as a function of time for unclear reasons. TEWL increased 23% from D0 to D1 (3.34 to 4.11 g/m^2^h), 34% from D0 to D3 (to 4.11 g/m^2^h) and 31% from D0 to D7 (to 4.37 g/m^2^h; <0.02 for these comparisons, 2-tailed *t *tests). There were not significant differences in TEWL from D1 to D2, D1 to D3, D1 to D7, nor D1 to D28 (*p*> 0.12, 2-tailed T-tests). Due to changes in control TEWL from baseline, subsequent statistics were adjusted for changes in time-matched control skin.

SLS profoundly increased TEWL. Combining pre-treated (sites to be treated with Delta-5) and untreated SLS-damaged groups together (*n* = 14), TEWL increased 7.8-fold from 3.47 at D0 to 27.19 g/m^2^h at D1 (*p* = 0.0002). The *p*-value was ≤ 0.01, with groups separately (*n* = 7). Technicians were instructed to apply Delta-5 to more damaged sites (Site 3). D1 TEWL was 57% higher in pre-treated sites than untreated sites (33.23 avg vs. 21.16 g/m^2^h, respectively; *p* < 0.02).

### TEWL, impedance-Delta-5 effects

**Fig. 3:**
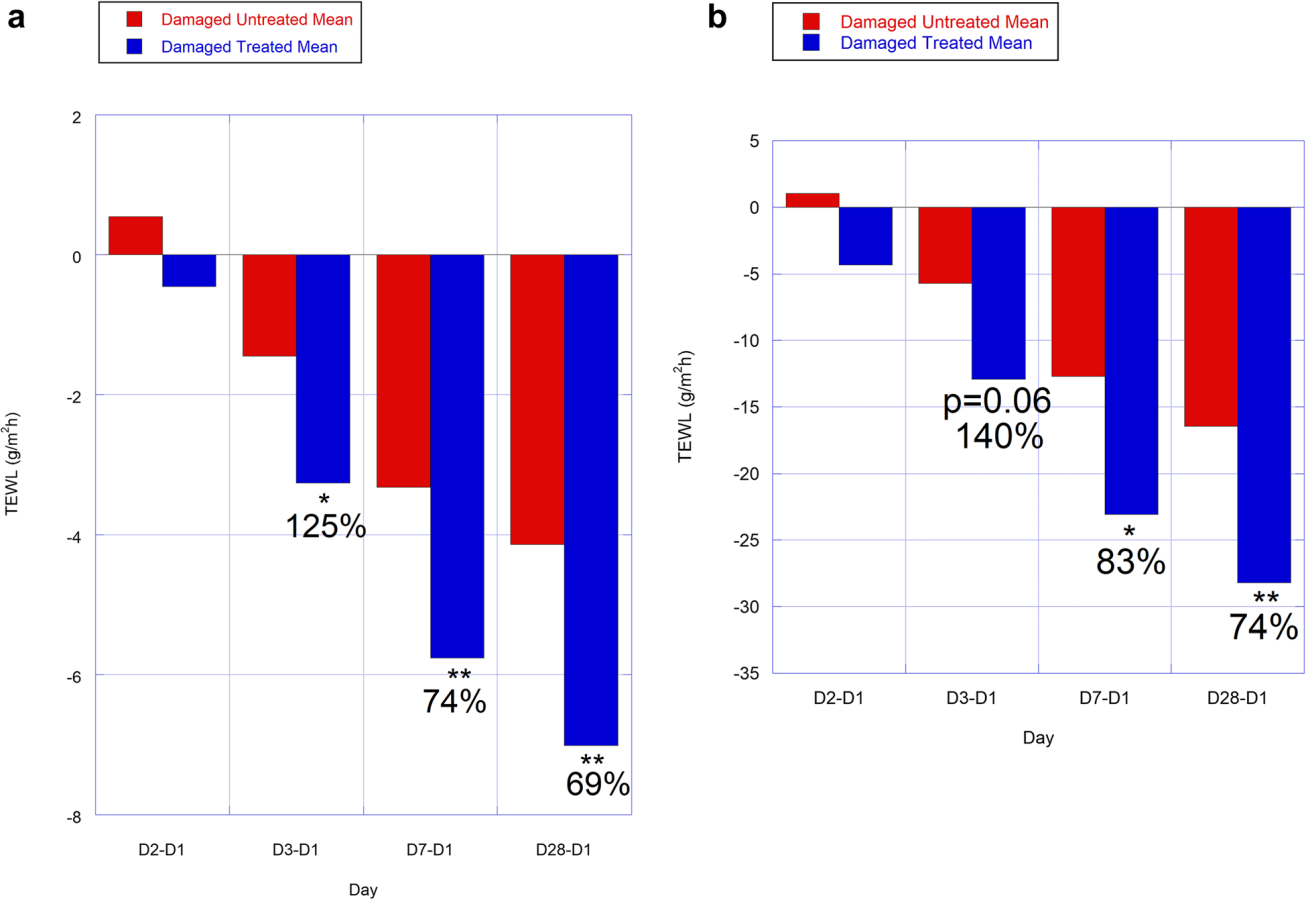
**a** and **b**: TEWL (g/m^2^h) evaluated as differences from Day 1 in Delta-5 oil-SLS damaged and untreated damaged groups at various time points, with- (Fig. 3a) and without (Fig. 3b) control correction. Values represent the means of differences from each time point from day 1. Values from each time point were divided by the corresponding untreated control value in Fig. 3a. *p ≤ 0.05; **p ≤ 0.01. Percents displayed represent the percent differences between Delta-5 oil-SLS damaged and untreated damaged groups. Standard deviations are not shown for readability of the graphs; and can be found in Table 1

Relative to D1 TEWL (normalized by time-matched control values), Delta-5 decreased TEWL vs. untreated SLS sites, on D3 (125% more reduced; *p* = 0.03), D7 (74% more reduced; *p* = 0.01), and D28 (69% more reduced; *p* = 0.006; Table 1; Fig. 3a). Differences were not significant for this statistic at D2 (means ± SD were − 0.5 ± 4.1 for Delta-5-; and 0.5 ± 3.2 g/m^2^h for untreated- SLS-damaged sites; *p* = 0.23). Without normalization by time-matched control values, the same trends as above were observed relative to D1 values (*p*-values were 0.06, 0.02, and 0.01 for days 3, -7, and − 28, respectfully; Table 1; Fig. 3b). With a small sample size of *n* = 7, it is particularly important to evaluate individual subject responses. On D3, 71% of subjects (5 of 7) showed more improvement with Delta-5 than no treatment following SLS-damage. On days 7 and − 28, 100% of subjects (all 7) showed more improvement.

**Table 1:** TEWL and Impedance evaluated as differences from Day 1 in Delta-5 oil-SLS damaged and untreated damaged groups at various time points

Time points/Means	D2-D1	D3-D1	D7-D1	D28-D1
	**TEWL With Control Normalization**			
Damaged Untreated	0.54 ± 3.23	-1.45 ± 3.37	-3.32 ± 3.51	-4.14 ± 3.47
Damaged Treated	-0.45 ± 4.09	-3.26 ± 3.98^*^	-5.76 ± 4.47^**^	-7.02 ± 4.68^**^
P-Value	0.24	0.03	0.01	0.01
% difference		125.2	73.6	69.4
	**TEWL Without Control Normalization**			
Damaged Untreated	0.84 ± 10.42	-5.14 ± 12.99	-12.46 ± 13.61	-15.86 ± 13.35
Damaged Treated	-4.53 ± 16.31	-12.36 ± 17.35	-22.80 ± 17.93^*^	-27.60 ± 18.52^**^
P-Value	0.17	0.06^Trend^	0.02	0.01
% difference		140.3	83.0	74.1
	**Impedance Without Control Normalization**			
Damaged Untreated	14.67 ± 84.85	-28.67 ± 119.75	-40.00 ± 118.30	-8.00 ± 98.20
Damaged Treated	-3.33 ± 100.20	-73.67 ± 148.65^**^	-82.00 ± 140.76^*^	-42.33 ± 145.19
P-Value	0.14	0.01	0.02	0.08^Trend^
% difference		157.0	105.0	429.2

Values represent means for differences from each time point from day 1 ± 1 SD for 6–7 subjects. Units for TEWL are g/m^2^h; units for impedance are in arbitrary Dermal Phase Meter impedance units (DPMIU). For impedance, subject 589,750 was excluded due to missing values and an outlying value (*n* = 6). Values from each time point were either normalized by the corresponding untreated control value for the corresponding time points, or not, as indicated. Asterisks are indicated as*p ≤ 0.05; **p ≤ 0.01, with p-values from 1-tailed, paired *t *tests also provided (see 17 section for details). Percents represent percent differences between treated- and untreated groups at each time point interval, and are provided when the *p*-value was between 0.05 and 0.10, as indicated as a statistical trend (“Trend”)

Residual skin damage is the amount of damage from SLS remaining at each time point studied. This calculation is typically presented in TEWL experiments but is crude in that damage is assigned a value of 0 on D0 and 100% on Day 1 (after the irritant is applied). It was specifically calculated for untreated and treated sites as follows: (D_x_/(D1-D0)) - (D0/(D1-D0)), where X = D2, D3, D7 and D28, D0 represents 0% damage, and D1 represents 100% damage. In untreated sites, residual damage was 102 ± 98% (means ± 1 SD), 46 ± 39, and 11 ± 6% at days 3, 7, and 28, respectively. In treated sites, residual damage was 78% ± 52%, 33 ± 25%, and 10 ± 12% on these same days, respectively. Only on D7, was a slight statistical trend (*p* = 0.09; 1-tailed, paired testing) observed for untreated and Delta-5 treated groups. By day 28, there was thus approximately 10% damage remaining (90% skin barrier repair) whether treated with Delta-5 or not, with this type of calculation.

**Fig. 4:**
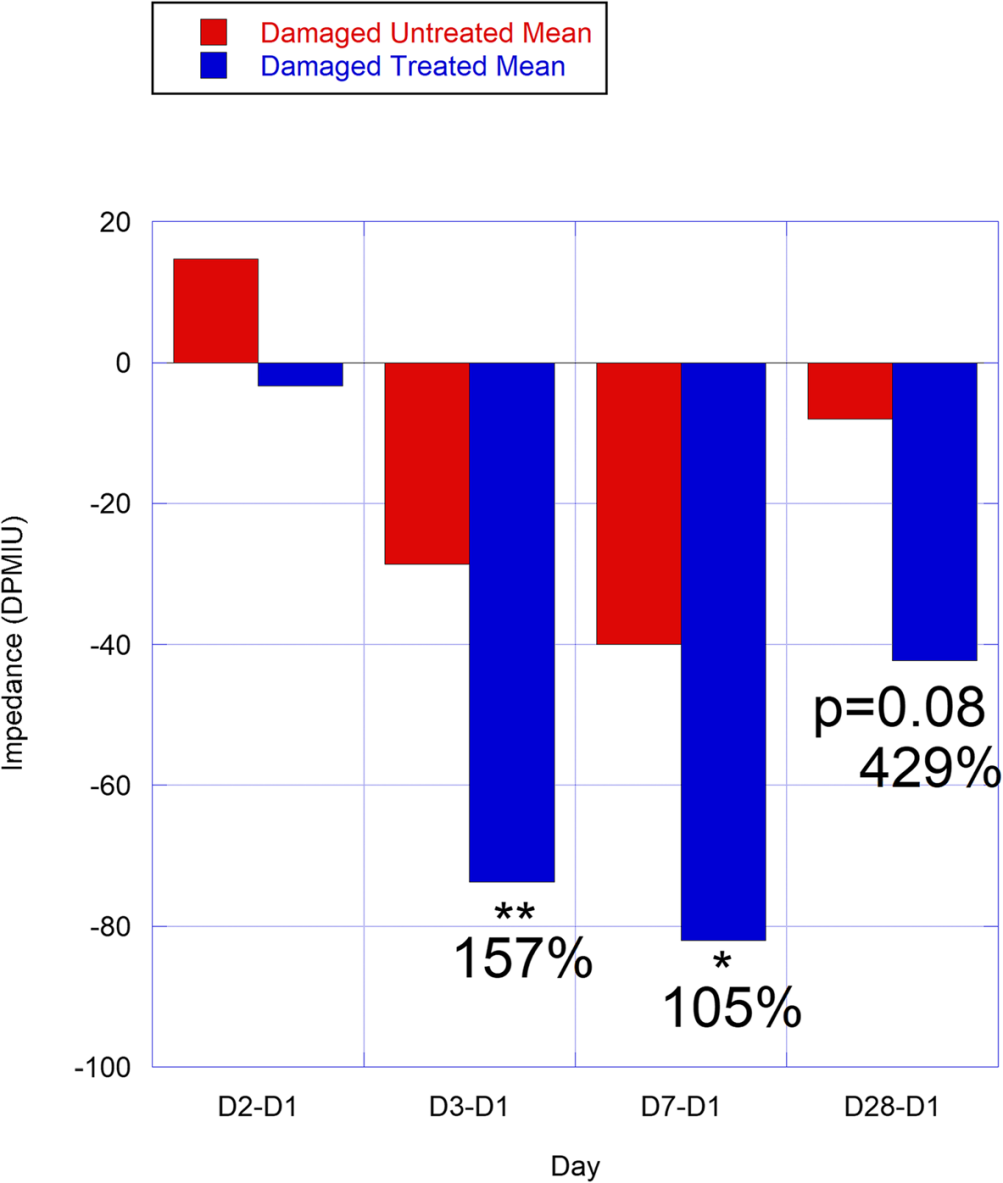
Impedance (Units are in arbitrary Dermal Phase Meter impedance units; DPMIU) evaluated as differences from Day 1 in Delta-5 oil- and untreated groups, shown as means at various time points. See Fig. 3 for statistical details and explanations. Standard deviations are not shown for readability of the graphs; and can be found in Table 1

Skin surface impedance showed the same trends as TEWL. When TEWL was reduced, there was also less water on the skin surface and lower impedance. There were missing values for one subject (58 9750). This subject also had a clearly outlying impedance value for control undamaged skin on Day 1 (a value of 3.00 vs. a mean of 114.7 ± 9.7 for the other 6 subjects) and was thus excluded. Skin impedance was not normalized to time matched controls as there was high variability in control values. This variability could be due to filling of superficial voids in the skin [28]. Relative to D1, Delta-5 decreased impedance vs. untreated SLS sites, on D3 (157% more reduced; *p* = 0.01), D7 (105% more reduced; *p* = 0.02), and D28 (429% more reduced; *p* = 0.08; Table 1; Fig. 4). Relative to undamaged sites (D0), D1 DPMIU values were higher as expected, but statistical significance was not reached due to high variation (*p* = 0.18 and *p* = 0.26 for untreated- and treated damaged groups, respectively). By D2, impedance reached its maximum, being 47% higher than D0 (*p* = 0.02–0.05); treated- and untreated SLS sites were not statistically significant from one another (*p* = 0.26).

### Skin redness

**Fig. 5:**
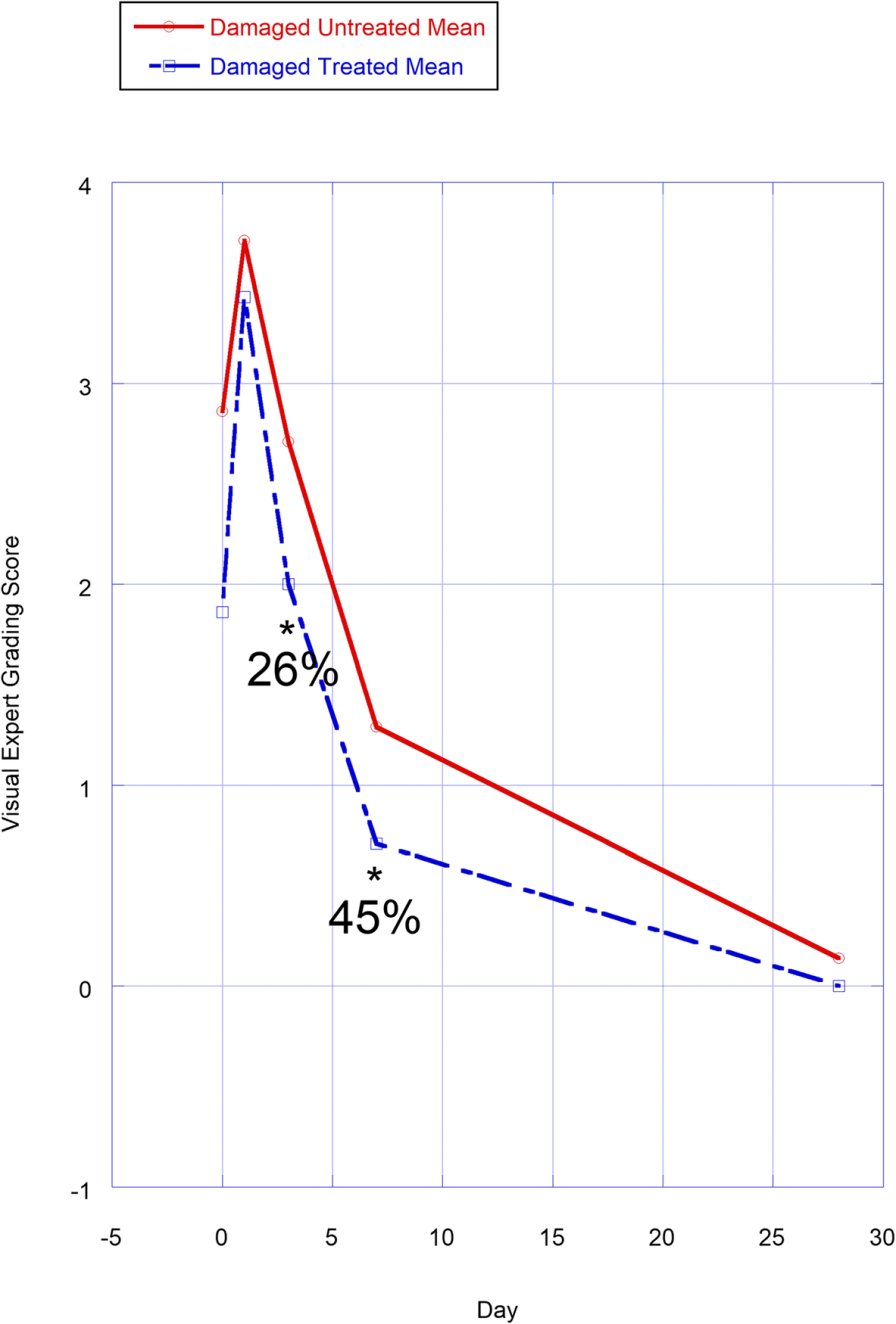
Redness (Visual Expert Grading Scores; VEGS) evaluated in Delta-5 oil and untreated groups at various time points, shown as mean VEGS at each time point. Note that redness was not evaluated as differences from Day 1 for reasons explained in the text. Percents displayed represent percent differences for the means between Delta-5 oil-SLS damaged and untreated damaged groups at each time point. Standard deviations are not shown for readability of the graph but are found in the section “Results SLS study-Skin redness”. *p ≤ 0.05

Redness was evaluated in untreated- and Delta-5-treated sites after SLS application. Mean redness scores at each time point are shown in Fig. 5. Redness was also evaluated as a categorical variable by coming several redness scores together (Table 2). Redness VEGS ranged from 0 to 7 throughout the 28 days. Immediately after SLS application (D0*), 13/14 sites showed low levels of redness (VEGS ≥ 1). D0* VEGS were 2.86 ± 1.68 and 1.86 ± 1.35 (means ± SD) for untreated- and pre-treated sites, respectively. D0* means were not statistically significantly different from one another (*p* = 0.14; 2-tailed *t *test), with combined D0* value of 2.36 ± 1.55 VEGS (14 sites). By D1, VEGS increased to 3.71 ± 2.29 and 3.43 ± 2.76 for untreated- and pre-treated sites, respectively, the maximal mean values observed for the time points assessed. D1 VEGS means were also not statistically significant from one another (*p* = 0.36, 2-tailed *t* test), with combined D1 VEGS of 3.57 ± 2.44. D3 VEGS decreased to 2.71 ± 1.98 and 2.00 ± 1.91 for untreated- and treated sites, respectively; and D3-treated sites showed a statistically meaningful 26% reduction in redness vs. untreated sites (*p* = 0.05, 1-tailed testing). On D3, highest redness category VEGS of 5–7 were observed in 29% of untreated- vs. only 14% of treated sites. On D3, all untreated sites showed some redness (VEGS ≥ 1); but 29% of treated sites were fully resolved (no redness; VEGS of 0). By D7, VEGS decreased further to 1.29 ± 0.95 and 0.71 ± 0.49 for untreated- and treated sites, respectively; and D7-treated sites showed a statistically meaningful 45% reduction in redness vs. untreated sites (*p* = 0.05, 1-tailed testing). By D7, a VEGS of 5–7 was not observed in untreated- nor treated sites. However, on D7, 29% of untreated sites still had an intermediate VEGS of 2–4 vs. no 2–4 VEGS with treatment. Moreover, on D7, redness was fully resolved in 14% of untreated- vs. 29% of treated sites. By D28, redness was fully resolved in all untreated- and treated sites, excepting a VEGS of 1 at 1/14 sites. There were no statistically significant differences between untreated- and treated sites on D3 and D7 after subtracting D0*- or D1-starting values (i.e., D3-D0*, D3-D1, D7-D0*, D7-D1), due to large variance in the combined statistics.

**Table 2:** Redness categorical data evaluated in Delta-5 oil-treated and untreated groups at various time points

Scores/Days	D0*	D1	D3	D7	D28
	**Damaged Untreated Score % of Categories**				
5–7	14	57	29	0	0
2–4	57	14	43	29	0
0–1	29	29	29	71	100
> 0	100	100	100	86	14
0	0	0	0	14	86
	**Damaged Treated Score % of Categories**				
5–7	0	43	14	0	0
2–4	57	14	43	0	0
0–1	43	43	43	100	100
> 0	86	86	71	71	0
0	14	14	29	29	100

Redness (Visual Expert Grading Scores; VEGS) were detected from a value of 0 (no redness detected) to a maximum observed value of 7. Categorical values represent the percentages of total subjects on each day within that range of scores. For example, on D0, 29% of subjects had a score of between 0–1, 57% had a score between 2–4, and 14% had a score of between 5–7, totaling 100%. If the total is slightly more than 100%, this is due to rounding errors. The last two rows for untreated and Delta-5 treated, show the groupings as 0 (no redness detected), or > 0 (redness detected). No statistics were applied to this type of data. D0* represents the measurement of redness immediately after application of SLS at baseline. D1, D3, D7, and D28 represent the days after application of SLS. Treatment with Delta-5 oil began just after D1, so D3 represents 2 days treatment with Delta-5. No redness measurements were made on D2

## Discussion SLS study

### Overall findings

Delta-5 oil, containing 24% of the bioactive, unique fatty acid SA, was extrapolated to have a stability of at least one year at room temperature in a neat formulation once opened, on basis of accelerated shelf-life testing and empirical findings. Delta-5 was also not a contact sensitizer nor skin irritant basis RIPT testing. Delta-5 oil thus has suitable stability and safety for cosmetic usage. In terms of efficacy, Delta-5 likely improved healing of SLS irritant-induced skin injury basis decreases in TEWL, impedance, and redness (vs. untreated skin) on days 3, 7 and 28 (D28 for TEWL and impedance). Delta-5 may thus benefit skin diseases such as dermatitis and eczema; and in normal skin, may improve skin elasticity and firmness.

TEWL and impedance showed consistent responses for evaluating skin barrier repair in response to SLS damage herein, and as reported by others [17, 24]. With both methodologies, Delta-5 accelerated healing following SLS damage over untreated skin at days 3, 7, and 28.

SLS damage from 2% SLS was maximal on days 1 and 2, basis increased TEWL and impedance. We did not test for reactive hyper-hydration (acute TEWL- and impedance increases) potentially occurring immediately after SLS-exposure [29]. Other groups have similarly reported greatest increases in TEWL and skin surface water 1–2 days after SLS, with repair commencing on days 3–7 [29–31]. SLS-damage was expected to be fully repaired by 28 days, but we found skin was not fully healed in all subjects at 28 days, based on TEWL. Similar to Delta-5, ceramides were also reported to promote healing from SLS after even 28 days [32], suggesting benefits of longer term usage following an acute injury or irritant insult. Others have reported TEWL to be elevated after 1–2% SLS for 9–14 days [29, 31], the last time point measured.

The greater reduction in redness on days 3 and 7 with Delta-5 vs. untreated SLS-damaged sites indicates a redness-reducing benefit temporally coinciding with improvements in TEWL and impedance during the healing process. Reductions in redness following Delta-5 usage is one of the most common customer testimonials reported. There was high variability in redness scores at all time points across individuals, suggesting differential development- and resolution of redness. Redness may have been more uniform had forearms been cleaned with gentle soap prior to SLS-application. Similar to our results, in experiments with 1–2% SLS given to healthy volunteers for 24 h on the forearm, changes to TEWL and skin redness persisted 7–14 days [29–31]. In the future, more sensitive methods for assessing redness could be employed, including erythema-index, utilizing a hand-held spectrometer [33].

### Proposed mechanisms of action for improvement in barrier function

The amount and type of skin surface fatty acids may affect barrier function as assessed with TEWL following skin irritation damage [34]; and have a role in the etiology of skin barrier dysfunction in diseases such as atopic dermatitis [35]. We will explore below how topical application of SA could affect barrier function repair via proposed structural and signaling roles.

#### Alteration of lipids in the extracellular spaces of stratum corneum

Permeability barrier of skin is mediated primarily by lipid-enriched lamellar membranes localized to extracellular spaces of stratum corneum. This unique structure contains 50% ceramides 1–6 (ceramides 1 and 3 being most important in barrier function disease etiology), 25% cholesterol, 15% free fatty acids, and very little phospholipid [13, 14, 36–40]. Delta-5 oil could affect ceramides, free fatty acid levels, and phospholipid acyl composition in the lamellar membrane [1]; and this in turn could affect skin impedance and TEWL [41, 42]. Of the fatty acids in Delta-5, linoleic acid (42% of fatty acids detected in Delta-5), palmitic acid (4%), stearic acid (2%) and 20:1n-9 (2%) could be incorporated into structural ceramides. Linoleic acid would only be incorporated into ceramides 1 and 4, linked to an omega-hydroxy fatty acid [43]. As SA is not incorporated into sphingomyelin, the sphingosine base being common to sphingomyelins and ceramide [44], we would not expect SA to be a component of ceramides.

#### Effects on the free fatty acid pool and stratum corneum pH

Following skin surface lipase action on the triacylglycerols present in Delta-5, the free fatty acid pool generated could affect the acidic pH of approximately 4–5.5 at the stratum corneum surface and influence phase behavior [43].

#### Incorporation into keratinocyte phospholipid membranes

Fatty acids in keratinocyte phospholipid membranes are either synthesized endogenously or derived from extra-cutaneous sources [37]. Indeed, SA and other fatty acids in Delta-5 including linoleic acid (42%) and potentially its 2-carbon elongation product 20:2n-6 (11%) via retroconversion may be incorporated into keratinocyte membranes [1]. Increases in lipid synthesis and lamellar body secretions are generally accepted to improve skin barrier function and permeability [45].SLS alters the repair phase of human skin via alteration of keratinocyte differentiation markers, and changes in enzymes degrading corneodesmosomes (intercellular adhesive structures in the stratum corneum) [46]. Following SLS-damage, fatty acid transport is increased by epidermal cytosolic fatty acid binding proteins [47], increasing fatty acids such as those present in Delta-5 in extracellular spaces.

#### Anti-inflammatory signaling roles of SA

Upon release from cell membrane phospholipid pools, SA may also have important anti-inflammatory signaling roles. In cultured human skin keratinocytes, SA was not only incorporated into keratinocyte membranes, but also reduced levels of pro-inflammatory ARA and its pro-inflammatory down-stream mediator prostaglandin E_2_ (PGE_2_) [1]. When SLS was applied to human skin *in vivo*, it induced differential expression of cyclooxygenase-2, the enzyme involved in synthesis of PGE_2_ [48]. Fatty acids such as SA can also affect gene transcription; and bind to receptors in cell signaling cascades [49–51]. In particular, the peroxisome proliferator-activated receptor PPARγ is activated by some fatty acids including linoleic acid present in Delta-5 oil, leading to anti-inflammation in skin [47]. In irritant contact dermatitis, PPAR agonists accelerate barrier recovery and enhance lamellar body synthesis, and neutral lipid synthesis (ceramides, cholesterol) [47]. Our earlier work with keratinocytes demonstrated however that at least unmetabolized SA does not bind to PPAR receptors [1]. SA could also have cannabimimetic actions that affect skin inflammation and healing [52–54].

#### Delta-5 oil may have barrier repair emollient and humectant properties

As a highly spreadable, cosmetic-grade, refined oil, Delta-5 could act as a barrier repair emollient and have humectant (moisturizing) properties, independent of the SA content. Emollients can decrease pro-inflammatory cytokines in skin with damaged epidermis [55], so it is feasible that the oil components of Delta-5 and SA both act to improve barrier function and barrier layer recovery from injury and irritation. To test the specific biological activity of SA would require that another skin site be treated with purified SA, or with a control oil with all fatty acid components equivalent to those in Delta-5, excepting the SA content. In rodent oral feeding studies, we demonstrated that it was the SA component of conifer oils rich in SA that displaced ARA from lipid pools, utilizing such an approach, exerting anti-inflammatory properties [44, 56]. Moreover, purified SA has been demonstrated in various models, including skin models, to be biologically active and anti-inflammatory on its own [1].

### Delta-5 oil vs. other therapies to improve barrier function

An advantage of SA over steroidal and non-steroidal anti-inflammatory agents, and other drugs, for treating diseases with barrier layer dysfunction is that SA usage should not result in any tapering of benefits, and there should not be any adverse side effects (none observed amongst customers of Delta-5 oil and in pre-clinical studies to date). Topical glucocorticoids may lead to detrimental decreases in levels of stratum corneum lipids, countered by ultraviolet light [43]; there is no reason to expect therapies with SA should have this negative effect.

Most cosmetic products developed for improving condition of the barrier layer act as emollients and humectants. As previously noted, Delta-5, may share these beneficial properties, with the additional benefit of SA acting in specific pathways to decrease inflammation associated with barrier layer dysfunction. Delta-5 would likely be even more effective if penetration enhancers or emulsifiers were added in an oil-in-water- or water-in-oil emulsion. An emulsion would additionally provide oxidative stability to the labile components in Delta-5 oil, such as SA.

Delta-5 oil (which consists of triacylglycerols) may not penetrate skin surfaces optimally, unless the skin surface is damaged, as in SLS-damaged skin. There must also be sufficient skin surface lipase activity from the host or microbes to catabolize the triacylglycerols in Delta-5 oil. We observed that Delta-5 penetrated the skin more efficiently following electroporation with a portable ultrasound device (Unpublished results). Another strategy to improve penetration would be to administer topical SA or oils rich in SA in the form of ethyl esters. Ethyl esters are approved for cosmetic use, commercially feasible to manufacture, and oxidatively stable. In previous work, we administered the related methyl esters of Delta-5 to mouse ears, and demonstrated penetration of SA and other fatty acids into the mouse ear skin; Delta-5 also reduced edema in the model system [1].

### Systemic benefits of Delta-5 oil

Epidermal and skin barrier layer dysfunction can lead to development of chronic, low-grade systemic inflammation and inflammation associated with aging (so-called “inflammaging”) and associated systemic disorders, particularly in aging [57, 58]. Correction of epidermal dysfunction with topical Delta-5 could thus be valuable for ameliorating aging-associated systemic disorders. As SA also has anti-inflammatory benefits when consumed orally based on rodent models, a combined strategy consisting of topical- and oral SA could be particularly beneficial for combatting inflammaging. It will also be valuable to determine if SA/Delta-5 oil effects the vast cutaneous microbiome, perturbations of which can affect inflammation [4].

### Strengths and limitations

Delta-5 oil was well characterized for its fatty acid composition and stability; and then determined not to be a contact irritant. The product was then evaluated in the first formal controlled clinical testing. A strength of the study was that results were validated, and showed consistency, by testing both TEWL and impedance, along with redness, by an experienced team of investigators using validated methods and equipment. The parameters were assessed until 28 days showing long term benefits of Delta-5; whereas in most clinical trials using SLS as an irritant, TEWL, impedance, and other parameters are measured for only 7 days [29, 30].

### *Limited sample size*

This was a first pilot clinical trial to test the effects of Delta-5 in an established irritant-induced skin barrier damage model against non-treated damaged sites. Our testimonial data combined with an understanding of the mechanism of action of sciadonic acid acting as an anti-inflammatory fatty acid, suggested this could be a good first pass skin model system to evaluate if Delta-5 would generate statistically- and clinically meaningful results. We did not know a priori what sample size would be appropriate, but the data obtained herein, will be useful for future sample size calculations in more elaborate, future studies. As a first pilot study, our strategy was to maximize potential to observe statistically significant results, albeit with caveats including a limited number of subjects (*n* = 7) who were not diverse (Caucasian females aged 45–58). In males, and persons with a tougher skin surface, there would possibly be less damage with SLS, but we would still expect Delta-5 to be effective; this would need to be tested empirically. In addition to gender and skin toughness, skin tone or fairness is an example of a factor increasing TEWL following SLS damage [59, 60], not recorded herein, that could be considered in future trials. Elevated levels of stratum corneum cytokines, age, and anatomical site are other factors affecting TEWL values [18, 61].

With our relatively small sample size of *n* = 6–7, this increases the likelihood of Type II errors (concluding there were not statistical differences when there in fact were); but the magnitude of changes combined with relatively small variability, permitted for statistically significant conclusions to be reached. Our p-value was sufficiently small (*p* < 0.05) so risk of Type I errors (concluding there were statistical differences when there were not) was minimized. Even with the small sample size employed, we observed consistent improvements with Delta-5 vs. no treatment in damaged skin over the same time points, using three different instrument methodologies (TEWL, Impedance, and redness), adding overall confidence to our conclusions.

A more expansive sample size could be employed in subsequent trials to have statistical power to detect possibly smaller differences between Delta-5 and an active control- or competitor product site; and a more diverse study population could be employed. Assuming maximal differences on day 7, normal distribution, 5% alpha, 80% beta (power), 1-tailed testing (double the n for 2-tailed testing), SD of 2.09 (from repeated measures ANOVA), and maximal difference (d) of 1.22 (half the d-value for Delta-5- vs. untreated damaged sites), the total number of subjects completing a future trial would be 28 (PASS 2019 Power Analysis and Sample Size Software (NCSS, LLC, Kaysville, Utah). Allowing for 10% drop out rate, 31 subjects would initiate such a future trial.

### *Skin site selection bias?*

SLS was applied to two sites per subject on day 1 (24 h post-SLS application). Delta-5 was consistently applied to sites with more damage basis greater D1 TEWL values. This could introduce systematic bias. However, statistics for impedance and TEWL were calculated as differences from D1 (D2-D1, D3-D1, D7-D1, D28-D1). In Delta-5-treated damaged sites, both D3 and D1 values (for example) would be higher, so differences might be equivalent had Delta-5 been applied to lesser-damaged sites. We also do not know the outcome had Delta-5 been applied randomly to the two sites; or deliberately to introduce potential variability (Subject 1: Delta-5-more damaged site; subject 2: Delta 5-less damaged site; subject 3: Delta-5-more damaged site, etc.). More subjects would need to be tested to understand effectiveness of Delta-5 to accelerate healing from irritation injuries of different severities; a statistical co-variable could be incorporated into models to account for severity of injury if a statistically significant factor.

### *Additional parameters to measure*

Herein, the primary objective was to measure changes in skin water content with TEWL and impedance; a secondary objective was to assess redness. In the future, it would be beneficial to assess changes in a broader spectrum of parameters to better understand the clinical potential and mechanisms of action of Delta-5/SA, including: erythema; skin surface pH; skin hardness; skin- and lipid thickness of the epidermis; lipid amounts and types (such as levels of SA in free- and esterified forms, and eicosanoids) from punch biopsies; and inflammatory markers (such as interleukins, tumor necrosis factor). In addition to these targeted approaches, un-targeted approaches such as transcriptomics and metabolomics would expand our understanding of how SA works and potentially lead to novel discoveries. Dose responses and kinetics with Delta-5 alone, and in emulsions on different skin types would be of practical and commercial importance.

## Conclusions

Delta-5 is a novel, efficacious new oil to be used alone and with other ingredients to improve condition of the all-important skin barrier.

Knowing Delta-5 has healing benefits for SLS-induced skin irritation, paves way for other irritation and barrier function studies. Preventative benefits could be determined by applying Delta-5 prior to injury [62]. Irritants could be evaluated alone or in combination with other substances including toxic plants, insect irritants, and the anti-acne ingredient benzoyl peroxide [63]. Additional conditions manifested by lipid barrier dysfunction could be probed including dermatitis, acne, eczema, and skin scarring. Delta-5 may also accelerate healing from burns, mechanical injury, environmental pollutants, allergens, and wounds (by accelerating wound closure and mitigating pain). By influencing barrier function, Delta-5 could also affect permeation of topical drugs and bioactive substances through skin outer layers. In normal skin, barrier function has roles in improving skin moisturization, structure, elasticity, firmness, frown lines and collagen production; and Delta-5 has a potential positive role to play as supported by relevant clinical testimonials.

## Data Availability

The data are not deposited on external sites.

## Abbreviations

ARA: Arachidonic acid
D: Day
Delta-5: Delta-5 oil
DPMIU: Dermal Phase Meter impedance units
RBDW: Refined, bleached, deodorized, and winterized
RIPT: Repeat Insult Patch Test
SA: Sciadonic acid
SLS: Sodium lauryl sulfate
TEWL: Transepidermal water loss
VEG: Visual Expert Grading
VEGS: Visual Expert Grading Scores
